# Investigating the effects of Hydroalcoholic extract of jujube fruit (Zizyphus vulgaris L.) on second degree burn wound healing in Balb/c mice

**Published:** 2015

**Authors:** F Vafaei, F Abdollahzadeh

**Affiliations:** * Department of Surgery, Imam Ali Hospital,North Khorasan University of Medical Sciences, Bojnurd, Iran; ** Young Researchers and Elite Club, Boukan Branch, Islamic Azad University, Boukan, Iran

**Keywords:** jujube (Zizyphus vulgaris L.), burn, burn healing, Balb/c mouse

## Abstract

**Background and Objective:** Two thirds of all accidents and injuries leading to death all around the world occur in developing countries like Iran. One of these accidents is burn that can have unpleasant effects on the individual’s body and soul. Skin wound healing is a process that happens as a result of coordination between tissues, cells, and different factors. The remaining inflammation and insufficient amount of vessel construction are among the most important causes of delayed wound healing. In recent years, jujube fruit (Zizyphus vulgaris L.) has been reported to have anti-inflammatory effects as a traditional therapeutic agent. Therefore, the present study was conducted in order to investigate the effects of jujube fruit extract on second-degree burn wound among Balb/c mice.

**Materials and Methods: **The present empirical-interventional study included 48 Balb/c mice weighing approximately 30 +/- 3 gr. After burn wounds of 1.5 cm2 were created and second-degree burns was affirmed by a pathologist, the mice were divided into four control groups; one treated with Vaseline, one treated with silver sulfadiazine ointment, one treated with jujube fruit extract 1%, and a control group.

**Results: **In treatment groups, 1 gr ointment containing hydroalcoholic extract of jujube fruit was utilized twice a day until complete recovery. Afterwards, the four groups were compared with regard to the wound area and histopathology. The collected data were analyzed through one-way ANOVA and Tukey tests by using SPSS software.

**Conclusion: **There was a significant difference between the intervention group and the Vaseline and control groups with regard to the percentage of wound recovery (P smaller than 0.05). The results of the study indicated that the jujube fruit extract could accelerate burn wound healing among Balb/c mice. It is recommended that further research is conducted on the effects of different doses of this medicine on laboratory animals and then on humans.

## Introduction

Burn is a type of injury to the skin or mucous membrane (like cover of the mouth, stomach, conjunctiva, and airways) caused by extreme heat, extreme cold, chemicals, and electricity and leads to damage of the skin surface and halts vital functions of the skin (e.g., prevents the transmission of infectious microorganisms, maintains fluid balance, and regulates body temperature) [**1**]. Burn wound can go beyond these and involve structures under the skin like muscles, bones, nerves, and blood vessels.

Burn is one of the major problems of public health all around the world and especially in developing countries [**2**]. Injuries in extensive burns not only threaten the patients’ lives but also have serious physical, mental, and economic effects for the individuals, their families, and society [**3**]. Burn injuries are the third cause of accidental death in all age groups and the second cause of mortality among age groups of up to 4 years [**4**,**5**]. In the USA, 2 million patients are provided with medical services related to burn injuries every year [**6**,**7**], and children account for approximately half of these patients [**8**]. Out of these patients, 500,000 need medical care, 70,000 are hospitalized due to severe injuries, and 6,000 die because of burn injuries [**6**,**7**]. In Iran, burn injuries also cause illnesses and a remarkable increase in the mortality rate especially among children. According to the studies conducted during 1995 and 1998 in Tohid Burn Center, Tehran, out of 3341 cases, 1454 individuals (43.5%) were children under 16 and their mortality rate was 16% [**2**]. Studies conducted in Tabriz indicated that burn is the third cause of hospitalization of children (66%) [**9**].

Nowadays with advances in critical care, there has been an increase in early surgical intervention, improvement in wound care, prescription of systemic or local antibiotics, and the number of surviving patients [**10**]. However, in spite of all these advances, there are still a lot of people who experience and die of such accidents every year [**4**,**6**]. Infection is the major cause of death among these patients [**11**-**13**]. Utilization of local antibacterial medications and quick removal of burned tissues can remarkably reduce the infection [**11**]. Partial recovery of burn wounds, long-term treatment, high costs of treatment, and secondary complications of burns need conducting research on burn recovery process and quick reconstruction process of epithelialization in burnt patients [**14**]. Wound healing is a recovery process happening after lesions of the skin and other tissues [**15**]. One of the goals in medicine is to heal wounds in a short time with fewer complications. Shortening the healing time is highly important due to the decrease in the probability of infection or complications and cutting costs [**16**].

Since the use of medicinal herbs has been common since ever, one or some herbs or herbal extracts have been used for the treatment of different diseases, and the miraculous role of some herbs in treating specific diseases has been proved, they are increasingly used in treating different diseases [**17**]. There are numerous herbs that are used in traditional medicine of different nations in order to heal wounds. Using jujube fruit as a therapeutic agent is common among the people of Chaharmahal and Bakhtiari Province, and its anti-inflammatory effects have been reported in recent years [**18**].

Jujube (zizyphus zizyphus) is a shrub with an average height of 10 meters. It is native from tropical regions and has glabrous oval small green leaves that fall in winters. The olive-like fruit of jujube has many medicinal properties. It is first green, and after ripening, it turns red and wrinkles. In Italy, it is consumed as flavored candies with evening tea. In Korea, China, and Taiwan, the sweet juice of jujube fruit is drunk. In some regions, it is used to make vinegar. In Africa, it is used to make cakes. Canned jujube and tea with jujube flavor are consumed all over the world. However, in Iran, Pakistan, and India, it is consumed in its dried form [**19**]. Jujube contains a lot of glaze, about 5% proteins, 4% vitamin C, and minerals. Aqueous extracts of jujube wood contain ziziphic acid and ziziphomatic acid [**20**]. Jujube is relaxing and anti-moodiness. In Chinese and Korean traditional medicine, it is used as a medication that reduces anxiety and strengthens the spleen, the stomach, and digestive system. Jujube refines the blood, removes toxins from the body, makes the skin clear, and prevents cardiac problems [**19**]. It is laxative, contains a lot of glaze, and softens the chest [**21**]. Consuming the pith causes wounds and cuts to recover quickly [**19**]. It also makes the teeth resistant against decay. Jujube tea contains an anticancer agent called saponin. It helps damaged tissues recover and strengthens the muscles [**22**]. Boiled jujube leaves are used to relieve sore throat and their extract to treat it [**23**].

The present study was aimed at highlighting a compound that is useful in healing injuries with minimum side effects and used in quick reconstruction of epithelialization process in burnt patients.

## Materials and Methods

**The method of preparing Jujube hydroalcoholic extract:** Jujube fruit (zizyphus zizyphus) was prepared from one of the herb stories of the town. After the kernels were extracted, the fruit was dried at room temperature and in shade. Afterwards, it was turned into powder by using the electric mill. Then, 75 gr of jujube powder was added to 30% water and 70% methanol alcohol, and after 72 hours, it was smoothed by using Buchner funnel. Afterwards, soxhlet and rotary apparatus were used to conduct the extraction process, which led to obtaining 145 gr of extract.

**The effect of other methods on burn healing:** In the present study, three local burn medications of sulfadiazine, Vaseline, and jujube fruit extract were compared against one another. Sulfadiazine has been used for more than a century and increases wound healing. However, due to its low penetration power, it is less effective in fighting against microorganisms that accumulate in tissue scars [**11**]. Petroleum jelly, mostly known as Vaseline, is obtained from refining heavy petroleum oils that are distillation remnants at 360˚C. Vaseline is a traditional medication that has been used for a long time, and its applications vary from softening the skin to being used as an oily substance and burn medication. It covers the burnt surface. It does not have the capacity to absorb wound exudates, has low permeability, and is only suitable for superficial burns. However, it is really cheap.

**Laboratory Animals:** In the present experimental study, 48 Balb/c mice weighing approximately 30 +/- 3 gr were selected. After they were anesthetized and burn wounds of 1.5 cm2 were created on their backs and the second degree burns were proved, the mice were divided into four equal groups; one treated with jujube fruit extract 1%, one treated with silver sulfadiazine ointment 1%, one treated with Vaseline, and a control group. The animals were kept in 22-25 degrees C, moisture of 50%, 12-hour cycle of dark and light, with normal diet, and in separate shelves.

**Conducted Tests:**The animals were anesthetized with intraperitoneal injection of mixture of ketamine 50 mg/kg and xylazine 5 mg/kg. In order to create the wounds, the back hair of the mice was shaved and the skin was completely cleaned and disinfected with alcohol. Afterwards, by placing a hot round metal surface of 1.5 cm2 on the fifth thoracic vertebra for 10 seconds, second-degree burn wounds were created. The burn day was considered as the zero day, and treatment was started on the first day. For each group, 1 ml of the prepared ointment was used twice a day on the wounds. In doing so, the wounds and their surroundings were completely covered with the ointments. Nothing was applied on the wounds in the control group. All the wounds were left without dressing and open. Microbiological tests indicated that the ointments contained no microbial agents.

After the creation of the wounds until full recovery, the wounds were photographed on the 1st, 7th, 14th, and 21st day of the experiment while the animals were anesthetized. Photography conditions were constant during the experiment. The wound area was exactly measured by using the taken pictures through Video Image Analysis Software, and recovery percentage was calculated by using the following formula:

Wound percentage = wound area on the 1st day/ wound area on the target day * 100

Recovery percentage= 100 – wound percentage

In a histopathological study, the resulting microscopic views of the samples were ranked based on the rebirth of epithelial tissues, the extent of fibrotic reaction, proliferation of the fibroblasts, edema, inflammation, and wound contraction; ranks 1 and 6 indicated lack of recovery and full recovery, respectively.

To make an overall comparison between the groups, first Kruskal-Wallis test was run, then the collected data were analyzed by using descriptive statistics tests, one-way ANOVA, and Tukey test through SPSS software.

## Results

In the present study, the burns were examined with regard to the healing criteria (i.e. the rebirth of epithelial tissues, the extent of fibrotic reaction, proliferation of the fibroblasts, edema, inflammation, and wound contraction). Afterwards, the four groups were compared regarding healing time, the results of which are presented in **Table 1** . The statistical analysis proved a significant difference between the groups with regard to their healing time. The results of the healing effects in groups using jujube fruit, silver sulfadiazine, Vaseline, and the control group indicated that healing results on the 21st day in the jujube group was better than the silver sulfadiazine and the control groups, and the difference was significant (p less than 0.05). However, the results of healing effects in the jujube group was not significant compared with the Vaseline group (p>0.05). On the 14th day, an apparent recovery of the wound in the jujube group was better than in the Vaseline group (p less than 0.05).

The results of the histopathological comparison of the mice indicated that on the 14th and 21st days the jujube group had a better regeneration of epithelial cells and a more extensive fibrotic reaction than the control group, and there was less bleeding at the burnt area. The fibroplasia process at the burnt area in these groups had more progress, and the results of Dunn post hoc test indicated that the wound recovery percentage in the jujube group was 1% higher than the control group (p less than 0.01) and the Vaseline group (p less than 0.001) (p less than 0.05) (See **Table 1**).

**Table 1:** Comparing the recovery percentage among the groups based on Dunn post hoc test

Groups	Rank Loss	P-value
Jujube Extract	3.6	P < 0.05
Sulfadiazine	-16.67	P < 0.05
Vaseline	-33.3	P < 0.001
Control	-36.67	P < 0.01

## Discussion and Conclusion

Burn wounds are among the latest recoverable wounds, and depending on the burnt patients’ conditions, finding natural substances that accelerate wound healing with few side effects can create a revolution in treating burn wounds [**21**]. Agents that reduce inflammation and cause disinfection affect the burn healing [**24**]. Studies indicated that jujube fruit contains 9 fatty acids, 2 saponins, a lot of vitamin C, and 7 phenolic compounds including caffeine, caffeine acid, epicatechin, feruic acid, rutin, pins acid, hydroxy benzoic, and chromogenic acid [**25**-**27**]. On the other hand, research indicated that fatty acids enhance collagen synthesis and accelerate wound healing by increasing the level of interleukin-6 [**28**]. Therefore, the presence of this type of fatty acid in jujube is a positive factor, for the same reason anti-inflammatory effect of olive oil is attributed to the presence of fatty acids, which can substitute arachidonic acid in cell membrane and reduce the necessary substrate for inflammatory enzymes. A portion of anti-inflammatory effects in jujube fruit can be explained in the same way.

The present study was conducted to specify burn wound infection based on clinical symptoms like fever, increased redness, or heat around the wound, inflation, tenderness, and any type of smell or secretion of the wound. The results of the present study indicated that on the 21st day there was a significant difference between the jujube and control groups in terms of burn wound healing. Moreover, the histopathological investigations on the samples showed that the process of covering cell restoration was better, the fibrotic reaction was more extensive, and the bleeding around the burn wound was less prominent. Fibroplasia process at the burnt area in these groups had more progress and edema and inflation were less highlighted.

Since jujube fruit contains a lot of unsaturated fatty acids, vitamins A and C, feulic acid, and carotene, and the therapeutic effects of these compounds in relieving inflammation, as it was observed in the present study, jujube extract, accelerates burn wound healing among Balb/c mice. Therefore, jujube can be used in ointment form as an effective medication in healing burn wounds. It is recommended that further long-term studies should be conducted in order to examine the therapeutic properties of jujube fruit of different density in order to achieve better results.
